# Recent Advances in Cancer Immunotherapy Delivery Modalities

**DOI:** 10.3390/pharmaceutics15020504

**Published:** 2023-02-02

**Authors:** Palaniyandi Muthukutty, Hyun Young Woo, Murali Ragothaman, So Young Yoo

**Affiliations:** 1 BIO-IT Foundry Technology Institute, Pusan National University, Busan 46241, Republic of Korea; 2 Department of Internal Medicine and Medical Research Institute, Pusan National University Hospital, Busan 49241, Republic of Korea

**Keywords:** drug delivery, cancer immunotherapy, CAR-T cells, nanoparticles

## Abstract

Immunotherapy is crucial in fighting cancer and achieving successful remission. Many novel strategies have recently developed, but there are still some obstacles to overcome before we can effectively attack the cancer cells and decimate the cancer environment by inducing a cascade of immune responses. To successfully demonstrate antitumor activity, immune cells must be delivered to cancer cells and exposed to the immune system. Such cutting-edge technology necessitates meticulously designed delivery methods with no loss or superior homing onto cancer environments, as well as high therapeutic efficacy and fewer adverse events. In this paper, we discuss recent advances in cancer immunotherapy delivery techniques, as well as their future prospects.

## 1. Introduction

Many strategies for inducing an antitumor response against cancers have been developed. Conventional treatment modalities include surgery, radiotherapy, and chemotherapy, each of which has had advantages and disadvantages with limited success in improving clinical outcomes. A thorough understanding of how tumors interact with the host immune system will aid in development of cancer therapeutics [1]. The tumor microenvironment is a complex and dynamic network of cellular and non-cellular matrix, a complex cluster of malignant cells, tumor stromal cells, extracellular matrices, blood vessels, immune cells, and signaling molecules, which influences the response to antitumor activity. They interact within TMEs in conjunction with some signaling molecules, cytokines, and chemokines in a sophisticated manner for the growth and metastasis of cells. Tumor cells construct an immunosuppressive environment to promote tumor growth and immune evasion mechanism through mechanisms such as inhibiting the Th1 cells, which in turn induces cytotoxic T cell differentiation and Th2 antagonistic response. Designing a cancer immunotherapy with appropriate delivery modalities will overcome the drawbacks associated with cancer [2].

Cancer employs a variety of strategies to evade the immune system, including delaying or even stopping antitumor activities. These immune-evading mechanisms overpower the natural immune system’s antitumor activity, and promote tumor formation, and metastasis. These mechanisms continue to evolve as cancer progresses, becoming more diverse and complex in late-stage malignancies. Various host factors that make up the immune system influence treatment outcomes, which can lead to disease progression or regression. Blocking these immune evasion strategies had led to the discovery of new strategies for strengthening the immune response against cancer [3]. Recent advances in cancer biology and anticancer immunity, most notably the identification of numerous key immunosuppressive pathways, have greatly aided this immunotherapeutic revolution. The 2018 Nobel Prize in Physiology or Medicine was awarded to James Allison and Tasuku Honjo for their “discovery of cancer therapy through inhibition of negative immune regulation.” Specifically, the Nobel Prize was awarded for the identification of immune checkpoints (i.e., cytotoxic T lymphocyte-associated antigen 4 (CTLA-4) and programmed death/ligand 1 (PD-1/PD-L1)), which led to the development of antibodies targeting these checkpoints for anticancer treatment [4]. Cancer immunotherapy has revolutionized cancer treatment. In contrast to chemotherapies and other treatments that directly destroy cancer cells, these medications aim to boost antitumor immune responses with fewer side effects [4].

Various strategies are employed by tumors to evade the immune system, such as downregulating antigen processing or presenting machinery (MHCI, proteosome subunit latent membrane protein 2 (LMP 2) and LMP 7, transport associated with antigen processing (TAP) protein and tapasin) as to not be recognized by T cells, which is the triggering point of recognizing and attacking tumor. Another strategy employed by tumors is to downregulate IFN signaling. which may evade antigen presentation and subsequently result in evasion from the immune system. Cancer cells also cause T cell exhaustion by increasing the PD-1 and PD-L1 expression by various inflammatory and oncogenic signals leading to immune evasion. Other immune suppressive modulators such as TGF-γ, IL-8, IL-10, VEGF can be secreted into the TME by tumor cells, which in turn suppress dendritic cell maturation and T cell functions. Tumor cells may suppress T cell function by manipulating the metabolic composition in the TME to wither its activity effectively [5].

**Figure 1:**
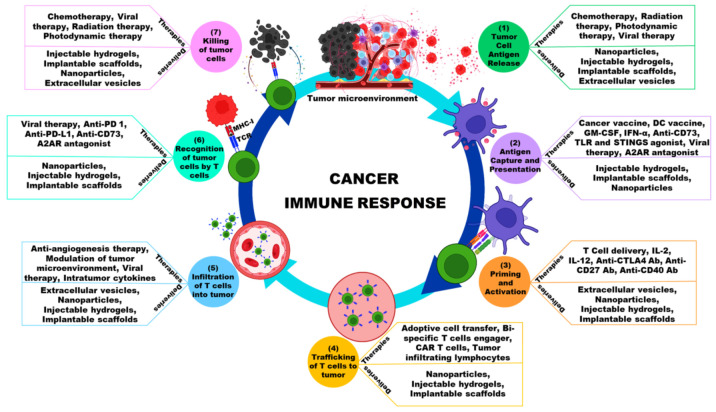
Schematic illustration showing the cancer immune response, interventional therapies and its delivery modalities.

The cancer–immunity cycle is a schematic representation of the principles of cancer immunotherapy. This cycle begins with the release of tumor antigens, which are taken up, processed, and presented to naive T cells (APCs). As a result, cytotoxic T cells are produced that can specifically recognize and kill cancer cells. Lysed cancer cells then release antigens and costimulatory signals, triggering another round of the immune response cascade. Tumors can disrupt critical elements of the cancer–immunity cycle via a variety of negative feedback immune regulatory pathways, which are increasingly becoming cancer immunotherapy targets [6]. These treatments aim to boost antitumor immune responses while having fewer side effects than chemotherapies and other drugs that directly destroy cancer cells. Therapeutic agents aiming to stimulate or increase the naturally ability of immune system to kill cancer cells, which often diminishes as the disease progresses, are used in cancer immunotherapy (Figure 1) [4]. Immunotherapy, which attempts to use the host’s adaptive and innate immune responses to achieve long-term eradication of diseased cells, can be broadly classified as passive or active [7].

The relationship between intestinal microbes and the immune system is mutual in developing tolerance against symbiotic bacteria and antigens present in the food. This makes the immune system prepare innate and acquired immunity against invading microorganism. There needs to be a balance between recognizing gut microbiota and invading pathogenic microbes. The metabolites produced by the microorganism in the gut can alter the balance of inflammatory cytokines in the body and disrupt the production of T cell subsets [8]. Recent research studies elucidate the relationship between gut microbiota and their function in cancer immunotherapy. In one of the studies with an immune checkpoint blockade targeting CTLA-4 and PD-1 using a mouse model, they showed that the gut bacteria have influence in the response tp cancer immunotherapy [9,10]. In another study, the ingestion of the bacteria *Bacteroides fragilis* with *Bacteroides thetaiotaomicron* or *Burkholderia cepacian* increased Th1 response and DC maturation, subsequently enhancing the efficacy of anti-CTLA-4 therapy [9]. Similar experiments conducted with immunotherapy using anti-PD-1 or anti-PD-L1 treatments showed the involvement of gut bacteria in modulating treatment outcomes [11,12,13]. In patients receiving PD-1 medication, the diversity of gut bacteria such as *Clostidiales*, *Ruminococcacease*, and *Faecalibacterium* are increased. Studies have found that the correlation between gut microbiota with respect to immunological profiling in the tumor microenvironment has demonstrated that cytotoxic T cell marker expression was augmented with antigen presentation and processing in patients having favorable gut microorganisms when compared to patients with unfavorable gut microbes [14]. Finding out the gut microbiota which have positive correlations with anti-cancer therapy can boost the efficacy and help patients to benefit from these therapies.

Immunotherapy is fundamentally changing the clinical cancer treatment landscape. It outperformed standard-of-care therapy in several cancer types, including malignant melanoma and lung cancer, resulting in a number of cases with remarkable outcomes, such as total regression of advanced-stage (metastasized) tumors and prolonged disease-free survival [15]. In addition to immune checkpoint inhibitors, which are primarily used for solid tumors, effective cancer immunotherapy has also been achieved through the use of chimeric antigen receptor (CAR) T cell therapies, which have thus far been primarily used to treat hematological malignancies [16]. In the last decade, the US FDA has approved several immunotherapy modalities for treatment, including five immune check point inhibitors, six CAR-T cell therapies, and one oncolytic virus therapy (Table 1).

## 2. Cancer Immunotherapy Types

### 2.1. Checkpoint Inhibitors

Check point inhibitors are the most extensively studied cancer immunotherapy modalities. CTLA4 inhibition and PD-1/PD-L1 blockade are the two most commonly used check point inhibitors. Check point inhibitors regulate the immune response to abnormal cells while protecting healthy tissues from immune attack [18]. T cells are activated to express PD-1 in response to inflammation in TME, which in turn make it possible to detect cancer cells [19]. Cancerous cells expressing PD-L1 render the T cells inactive by binding to them to avoid an immune response. Using checkpoint inhibitory monoclonal antibodies targeting PD-1 or PD-L1 to manipulate this phenomenon, T cells can be effectively used to counter cancerous cells [20,21]. CTLA4, a co-inhibitory molecule that regulates the T cell activation, has another check point inhibitor mechanism. The co-stimulatory molecule CD28 and its ligands CD80 and CD86 are important for the activation, proliferation, and survival of T cells. CTLA-4 blocks CD28 signaling by binding to its ligand CD80 or CD86 and thus inhibits proper T cell response. Thus, antibody against CTLA-4 is used as checkpoint inhibitor to activate T cells’ immune response [22]. The impact of PD-1, PD-L1, or CTLA4 checkpoint inhibitors has been regarded as one of the more efficient antitumor strategies than chemotherapeutics [23].

Cancer immunotherapy clinical trials are being planned in conjunction with check point inhibitors and chemotherapies or other agents [24]. Still, checkpoint inhibitors are not obsolete, and there are some limitations to them, such as severe side effects to certain organs when checkpoint inhibitors are systemically administered [25,26]. Still, clinical research studies are being conducted to determine the underlying mechanism of checkpoint inhibitors and their limitations in dealing tumor mechanisms [27].

### 2.2. Cytokines

Recombinant IFN was the first cytokine to be approved for immunotherapy in 1986. Since then, interferons, interleukins, and granulocyte macrophage colony-stimulating factor (GM-CSF) have been studied clinically for their immunotherapy potential [28]. Interferons are produced in response to microbial pathogen-induced immune activation, which results in the activation of macrophages, natural killer (NK) cells, dendritic cells, and lymphocytes. Interferon activates immune cells in the tumor microenvironment, inhibiting angiogenesis [29]. Interleukins stimulate the activity and growth of T cells, specifically CD4^+^ and CD8^+^ cells. There is a number of interleukins that have pro and antitumor activity (IL-4) depending on the tumor type, stage and location and type of cells which produce them. IL-2 is important for T cell proliferation, whereas IL-10 inhibits T cells activation [30]. GM-CSF stimulates the immune system through two mechanisms: it promotes T cell homeostasis and dendritic cell differentiation, which results in the production of tumor-specific antigens. GM-CSF can also help granulocytes’ recovery after chemotherapy [31]. Currently, extensive research is being conducted to reduce the adverse effects of individual therapies by combining checkpoint inhibitors with cytokines or chemotherapies [32].

### 2.3. Vaccinations

Tumor cell lysate, dendritic cells, and nucleic acids are examples of cancer vaccines [33,34,35]. Dendritic vaccines are the most extensively researched cell-based vaccines [36]. Autologous dendritic cells are collected from patients and engineered so that they express tumor-associated antigens, activating T cells to attack the tumor [37]. Sipuleucel-T is one of the approved dendritic cell vaccines for the treatment of prostate cancer that was approved in 2010 for its ability to successfully prolong patient survival [38]. Manipulating dendritic cells to express targeted antigens and induce T cells against tumor can improve the efficacy and potency of dendritic vaccines [39].

Nucleic acid therapeutics such as DNA- or RNA-based vaccines are emerging as alternatives to conventional vaccines [40]. Nucleic acid vaccines must be delivered intracellularly to the target cells, where they are translated to induce antigen expression. These antigens are presented to T cells in order to activate them against cancer cells. Recently, mRNA vaccines have gained attention because they have more advantages than DNA vaccines, such as the ability to extend the half-life of mRNA with minor modifications. However, mRNA is prone to degradation by nucleases, so it requires a transfection reagent or delivery technologies for intracellular delivery [41].

Vaccines are based on neoantigens that can boost the immune response against cancer cells [42,43]. Because of genetic instability, gene mutations occur in the coding region during carcinogenesis, resulting in the formation of proteins that are not present in normal cells. By activating the immune system, these proteins can be targeted specifically against cancer cells. Delivery methods must be designed in such a way that they increase the stability and protection against cancer [44].

### 2.4. Antibodies That Are Agonistic

Agonistic antibodies are specifically designed to bind to T cell receptors and activate intracellular signaling pathways in order to effectively combat cancer cells. Monoclonal antibodies (mAbs) targeting immune checkpoints such as CTLA-4 and PD1/PD-L1 have recently been developed for antitumor activity [45]. Agonist mAbs developed against the CD40 immune receptor can increase the tumor-infiltrating T cells (TILs), which can effectively eliminate cancer cells [46].When CD40 interacts with CD40 ligand in dendritic cells, it activates specific T cells, triggering a cascade of antitumor responses [47]. Agonist antibody-based clinical trials are currently being conducted against various receptors, targeting 4-1BB, OX40, and CD 27, but due to their toxicity, alternate delivery methods are required to mask their toxicity potential without compromising antitumor activity [48,49,50,51].

### 2.5. T Cells with Alternations

Following successful clinical trials and FDA approval, T cell engineering has recently gained attention. Autologous T cells were collected from cancer patients’ blood and genetically engineered to express chimeric antigen receptors found on tumor cells but not on healthy cells in the CAR-T cell approach. CAR-T cells recognize the target antigen on tumors and induce tumor cell death when re-engineered T cells are infused back into the patient [52,53]. The advantage of CAR-T cell therapy is that it is a single infusion therapy that can provide protection for up to a decade after injection [54]. CAR-T therapy has its own drawbacks, such as the fact that it is technically complex, time-consuming, and expensive to produce, which has been a concern in the implementation of CAR-T-based therapies [55]. CAR-T cells were unable to penetrate and interact with antigen receptors in certain solid tumors and complex TME, necessitating the use of combination therapies to improve the efficacy of CAR-T cell therapy [56,57].

Since 2017, the US FDA has approved six CAR-T therapies for blood cancers such as lymphoma, certain leukemias, and, most recently, multiple myeloma. CD-19 is a target antigen for B cell acute lymphoblastic leukemia (ALL), B cell non-Hodgkin lymphoma, follicular lymphoma, mantle cell lymphoma (MCL), and B cell maturation antigen (BCMA) targeting against multiple myeloma [58]. T cell receptor (TCR) T cells are T cells isolated from patients and genetically engineered to express specific peptides and human leukocyte antigens (HLA), resulting in TCR-T cells that are recognized by tumor-associated antigens and effectively kill tumor cells [59]. TCR-T cells, unlike CAR-T cells, are MHC-dependent, so they must be matched with the patient after genetic engineering, which is critical in TCR-T cell cases [60]. Both CAR-T cells and TCR-T cells require further development to improve their applicability with solid tumors while minimizing the associated side effects and toxicity.

### 2.6. Virotherapy with Oncolytic Agents

Oncolytic viruses have shown promise in the treatment of cancer. Specific viruses that can replicate in the cancer cells induce antitumor immune attacks in the tumor [61,62]. Viruses have been genetically modified to attack and destroy tumor cells while leaving normal cells alone [63,64,65]. Oncolytic viruses used against cancer immunotherapies include adenovirus, vaccinia virus, herpes simplex virus, measles virus, Reo virus, Newcastle disease virus, Coxsackie virus, vesicular stomatitis virus, and Pseudovirus [66]. Antitumor enhancement is achieved in oncolytic adenovirus CG0070 by expressing GM-CSF against bladder cancer [67]. David Ruano et al. showed that the combined treatment of oncolytic adenovirus ICOVIR-5 with mesenchymal stem cells resulted in disease stabilization in neuroblastoma patients, according to a first-in-human and child study [68]. Kim et al. studied several genetically modified vaccinia viruses. In a liver and lung model, deletion of thymidine kinase and expression of GM-CSF prevented metastases [69]. Yoo et al. demonstrated that a vaccinia virus lacking thymidine kinase effectively suppressed stem cell-like colon cancer cells [70]. In addition, they demonstrated that the engineered vaccinia virus can effectively eradicate metastatic liver cancer cells in another study [71]. T-VEC, an oncolytic herpes simplex virus engineered to secrete GM-CSF, was recently approved by the US Food and Drug Administration to treat advanced melanoma [72].

## 3. Administration Mode

The route of administration (ROA) of a drug can affect its therapeutic efficiency during the delivery process [73,74,75] ROA is an important consideration when developing the delivery immunotherapy delivery methods for a specific tumor treatment. When compared to non-target routes of administration such as systemic administration, directly injecting drugs into tumors (intratumoral) can elucidate better efficacy in terms of antitumor effect [76]. Intratumoral injection directly into the tumor is possible for accessible tumors, but for tumors that are not easily accessible, other modes of administration must be used to effectively deliver drugs to the tumors (Table 2) [77]. The therapeutic efficacy is proportional to the control-release mechanism, which affects how the payload drug is transported in the appropriate medium to comprehend the microenvironment. Understanding the tumor’s microenvironment and the accessibility for the drugs in order to effectively deliver the drugs is a challenge that must be considered. Innovative technologies for effectively delivering drugs for cancer immunotherapy are being developed.

## 4. Cancer Immunotherapy Delivery Methods

**Figure 2:**
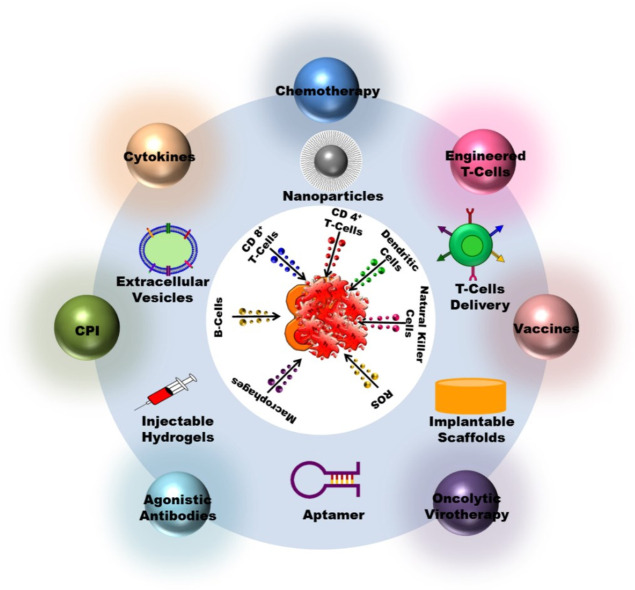
Efficient and effective delivery methods specific and less toxic to host cells used in cancer immunotherapy.

Successful cancer immunotherapy necessitates efficient and effective delivery methods, as well as drug efficacy that is specific and less toxic to host cells (Figure 2). The difficulties in treating a cancer with a drug or biomaterial range from dosage, formulation, homing, degradation, and delivery, all of which must be taken into account when designing a treatment. When developing a drug and its delivery methods, biological and physiochemical parameters should be taken into account. This section discusses the various methods for delivering effective cancer immunotherapy treatments (Table 3).

### 4.1. Nanoparticles

Nanomaterials are advantageous in several parameters, including surface-to-volume ratio, photo dynamics, magnetic and electrical conductivity, optical absorption, and fluorescent behavior properties, which make them an effective additive in cancer immunotherapy [122]. Recent technological aspects of nanoparticles have sparked interest in the use of nanomedicine-based drug delivery systems because they can potentially cross biological barriers, have biocompatibility, drug transport, and provide sustained drug release in cancer immunotherapy approaches [123]. To overcome the barriers to drug deliver to the tumor microenvironment, a powerful delivery platform that penetrates the complex structure surrounding the tumor is required [124]. The use of nanoparticles in drug delivery is one of the promising novel methods in the application of cancer immunotherapy [125].

Nanoparticle-based approaches to drug delivery drugs to tumors have attracted the interest of researchers because they are cancer cell target specific [126]. Nanoparticle-based delivery that directly targets the tumors can improve drug biodistribution and localization within the tumor [127]. Small molecules, proteins, peptides, antibodies, cytokines, and monoclonal antibodies can be delivered by nanoparticles using a variety of platforms, including liposomes, polymers, inorganic nanocarriers, dendrimers, and exosomes [128]. One of the most important properties for a nanoparticle in cancer immunotherapy is enhanced permeability and retention (EPR), which determines the drug’s accumulation time in the tumor microenvironment [129]. The use of tumor-associated antigens (TAA) to direct the immune system against cancerous cells results in less antitumor activity. Combining them with a nanodelivery system effectively protects them from degradation and allows them to interact with antigen-presenting cells, resulting in the stimulation of cytotoxic T lymphocytes with an effective antitumor mechanism [130].

Deng et al. used NK cell-masked nanoparticles, which can be activated by photodynamic therapy to attack the cells and induce immunogenic cell death (ICD), releasing damage-associated molecular patterns, as well as NK-coated cells targeting M1 macrophages, which eventually promotes antigen-presenting cells’ (APCs) maturation, leading to T cell activation and elimination [120]. Cancer cells were eradicated using novel photoimmunotherapy-based nanoparticles. By synthesizing an apoferritin nanoparticle protein cage as a photosensitizer conjugated with fibroblast activation protein specific antibody, Zhen et al. were able to effectively bind the fibroblasts in the tumor region, photoirradiation modulated the antitumor immune response [131]. Another strategy for inducing immune system against cancer is to manipulate nanoscale bioconjugates. One such strategy is the use of Halloysite nanotubes coated with polyethylene glycol, functionalized with folate residues and loaded with doxorubicin, a chemotherapeutic drug against 4T1-bearing mice, which demonstrated 65% tumor growth inhibition compared to 35% for doxorubicin alone [132].

Copolymer micelles have been shown to have EPR effects when used to target tumors. Grafting polylactic acid onto carboxymethyl cellulose as a copolymer and functionalizing with anti-EpCAM antibody can be used for doxorubicin chemotherapeutic drug delivery against hepatic cells (HepG2). Doxorubicin drug release was in specifically at the tumor site, and functionalized drug-loaded micelles exhibited antitumor effects in both in vitro and in vivo conditions [133]. Chiang et al. demonstrated that a combination of anti-PDL1 checkpoint inhibitors and T cell activators conjugated to superparamagnetic iron oxide nanoparticles and functionalized fucoidan–dextran forming IO@FuDex^3^ nanocomplexes were capable of activating immune cells and neutralizing tumors in a 4T1 breast cancer mouse model [134]. Badrinath et al. demonstrated antitumor efficacy by enhancing apoptosis by combining an oncolytic vaccinia virus with poly lactic-co-lactic glycolic acid nanofiber as a delivery method against colon carcinoma [135]. Another method for targeting tumors is to use magnetic nanoparticles against tumors through various techniques such as manipulating the tumor environment, activating APCs, macrophage polarization, T cell stimulation, and NK cell delivery [136].

Nano vaccines are intended to contain tumor-specific antigens as well as TAA in order to suppress the tumor. Nano vaccines target antigens or components found exclusively in tumors or expressed in tumors. APCs such as macrophages and dendritic cells will come into contact with vaccine antigens [137]. Cell-, virus-, peptide-, DNA-, and mRNA-based vaccines have been shown to be effective in treating a variety of cancers. The main advantage of using nanoparticles is that they can be designed to produce an effective immune response against cancers based on the target cells [138].

Jin et al. demonstrated an in situ cancer vaccine-based approach; in their study, they designed in situ vaccines by combining two synergetic approaches. First, ferrimagnetic nano cubes were encapsulated into an amphiphilic polymer, which generates the antigens by a magnetic field and destroys the primary tumor, and another polymeric nanoparticle coated with adjuvant R848 (resiquimod) delivers the formed antigens to the lymph node, activates the APCs and creates an antitumor immune response to distant tumors [139]. Li et al. conducted another in situ based study. The formed TAAs were captured and delivered to APCs by photodynamic therapy, effectively eliminating cancer cells synergistically with checkpoint therapy [140].

In another study, fluoropolymer combined with antigen ovalbumin aided dendritic cell maturation and antigen presentation, leading to tumor suppression. When these fluoropolymers were combined with antigens from resected cell membranes from primary tumors, it resulted in inhibition of tumor recurrence and metastasis [141]. Luo et al. demonstrated the efficacy and abscopal effect of neoantigen-based immunotherapy against colon carcinoma and melanoma where nano vaccines inhibited tumor growth and survival rates in an in vivo model [142].

Organic and inorganic nanoparticles were combined in various ways to create effective photothermal agents to debilitate cancer cells [143,144]. Because of their biocompatibility and optical properties, gold nanoparticles were a candidate for nanoparticle synthesis, but their poor photothermal therapy application prompted a modification in their surface with silica; a silica-coated gold nanoparticle cluster was shown to displau effective photothermal transduction against prostate cancer cells in vitro and the tumors completely disappeared after 15 days [145]. Another method of inducing an immune response against cancer is to use nanometal organic frameworks (MOFs) loaded with anti-DEC205 antibody. Sonodynamic immunotherapy was used in this study, in which ultrasound-based deep-tissue-penetrating sonication functionalized the AMR-MOF@AuPt, resulting in large amount of reactive oxygen species that eliminated cancer cells and distant metastases [146].

Many strategies have been employed in attacking the TME using nanoparticles; one such strategy is to target the fibroblast cells associated within the tumor environment. Cancer-associated fibroblasts (CAF) were targeted using various nanoparticle delivery methods, and were able to be delivered into the deeper stroma reducing α-SMA (smooth muscle actin) levels around the tumor tissue and subsequently destroying the cancer cells [147,148,149,150]. Recently, studies have found that the macrophages are a double-edged sword; one could polarize macrophage into a tumor-suppressing subtype (M1) by exposing them to IFN-γ and lipopolysaccharides to produce IL-12, which arrests the tumor growth. Regulating tumor-associated macrophages (TAM) using iron oxide nanoparticles could polarize M2 macrophage into M1 macrophage [120,151,152,153]. One another strategy to target TME is by modulating the tumor extracellular matrix (ECM), as it provides support and regulates cellular activities and can be a targeting source to hamper the tumor growth using nanoparticles. Laminin in the ECM could be used as a target by designing laminin-mimicking, self-assembling peptides to form a nanoparticle, which would prolong retention time and accumulate at the tumor site and inhibit metastasis of cancer [154]. Other studies focus on components prevailing in ECM such as collagen, hyaluronic acid, matrix metalloproteinases, which are targeted using various nanoparticle techniques to suppress tumor growth and metastasis [155,156,157,158]. Nanoparticle-based strategies are used against vasculatures as they provide growth factors, nutrients and play an essential role in growth of the tumor; nanoparticles carrying anti-angiogenic drugs effectively inhibit angiogenesis and metastasis [159,160,161,162]. These nanoparticle-based strategies open new insights for cancer immunotherapy and can translate into clinical treatment for personalized therapy.

### 4.2. Vesicles Extracellular

Extracellular vesicles are small membrane vesicles formed by fusion of the plasma membrane and endosomes that are secreted by cells [163]. As everyday research reveals their potential in delivering drugs to cancer cells, EVs are emerging as a drug delivery technology [164,165]. EVs are complex membrane vesicles that travel through tight junctions to selectively enter cells [166]. Zitvogel et al. discovered the exosomes can be derived from dendritic cells with functional MHCs and tumor antigens on the surface, leading to tumor neutralization by cytotoxic T lymphocytes (CTL) [167].

Dendritic cell-derived exosomes increased NK cell antitumor activity in a clinical trial against non-small cell lung cancer (NSCLC) as maintenance immunotherapy for patients undergoing chemotherapy [168,169]. Wang et al. used exosomes to deliver drugs to a tumor in a liver mouse model. PTX, which has a low therapeutic efficacy due to its poor solubility, was packaged into exosomes to increase its potential and showed higher efficacy in tumor retention and inhibition [170]. Curcumin-loaded exosomes were used in another in vivo mouse model study to successfully cross the blood–brain barrier and deliver the drug against malignant glioma in the brain [171].

### 4.3. Biomaterials

Implantable functional scaffolds are frequently used in cancer immunotherapy to reprogram the biological responses by delivering bioactive chemicals or cells in a controlled manner [172]. Biomaterial-based delivery systems have properties such as minimal invasiveness, targeted delivery, controlled release, high efficacy, immune cell activation, and low toxicity, making them a potential cancer immunotherapy technique [135,173]. Nanomaterials and scaffold-based biomaterials are commonly used as implantable and injectable biomaterials to elicit immune responses and thus antitumor activity [174].

Long-term stimulation of APCs was achieved by constructing a 3D microporous alginate-reduced graphene oxide (rGO) scaffold loaded with GM-CSF, ovalbumin, and cytosine–phosphate–guanine oligonucleotides. The rGO component of the implantable scaffold’s large surface area and hydrophobic surface allow for significant loading and a very gradual release of a loaded antigen. In a B16 melanoma tumor model in mice, the scaffold recruited dendritic cells, which then activated T cells, effectively suppressing the tumor [175]. Another study found that loading a blood clot scaffold with liposomal nanoparticles containing both vaccine and siRNA can effectively induce DCs, leading to T cell activation and tumor suppression in various tumor and mouse models [176].

A cancer vaccine composed of whole tumor lysate-based antigens and nanoadjuvants expressing Toll-like receptor (TLR) 3 agonists, as well as gemcitabine as an MDSC-depleting agent, was shown to improve antitumor immunity by lowering immune suppression in the tumor microenvironment [110]. Similarly, Ren et al. used a degradable and regulatable macroporous implantable scaffold using methacrylate hyaluronic acid loaded with three different compounds, the chemotherapeutic medication PTX, APCs activator TLR7 agonist (R837), and immune checkpoint blockade molecules, which was then implanted in a 4T1 breast tumor mouse model, and showed depletion of myeloid-derived suppressor cells (MDSC) and M2 macrophages, enhancement of APCs, and increased antitumor immunity [177]. Ahn et al. created a 3D-engineered hyaluronic acid scaffold that increased mRNA expression, cytokine release, and tumor lysis, resulting in improved antitumor efficacy for a resected breast cancer model [112].

Because each type of biomaterial has distinct advantages in certain contexts, the choice of biomaterial design, whether injectable or implantable, is fundamentally driven by application requirements. Injecting implantable materials directly into the organs or tissues is a much less invasive procedure than surgical implantation, and it reduces the risk of tissue damage and the inflammatory response associated with wounds [172]. To make injectable biomaterials, hydrogels, cryogels, and self-assembling systems can be made from a variety of natural and synthetic ingredients [178]. Liu et. al, created a supramolecular hydrogel for locoregional delivery that functions as both an ICD and immune checkpoint inhibitor therapeutic [179].

In another study, they created an intelligent drug delivery system with controlled and sustained drug release. They created in an injectable nanofiber hydrogel by combining betamethasone phosphate and calcium ion with anti-programmed cell death protein ligand 1 antibody (αPDL1), which results in cross-linking filamentous assemblies. By blocking the NF-B signaling pathway, the anti-inflammatory steroid betamethasone phosphate has been incorporated into an injectable nanofiber hydrogel to reprogram the protumoral immunosuppressive TME, and the sustained release of PDL1 from the hydrogel stimulates the T cells to synergistically increase the immunological response of tumor cells [180]. Another strategy is co-delivery, which combines a hydrogel with a tumor vaccine and immune checkpoint inhibitors to improve the therapeutic efficiency against melanoma and 4T tumors [181].

### 4.4. T Cell Therapy Delivery Methods

The advancement of clinical grade bench-to-bedside technology for isolating, genetically engineering, and ex vivo expansion of T cells from one’s own patient blood has brought T cell-based therapies to the forefront of cancer immunotherapy [55]. Tumor-infiltrating lymphocytes (TILs) and T cell receptor manipulation results in expressing specific antigens and HLA to effectively eliminate tumor cells [182].

Adoptive cell therapy is one such T cell therapy that is effective in treating blood cancers. Chimeric antigen receptor-T cells (CAR-T) are one such therapy that has recently received several FDA approvals, and products in the US, for which patient blood is collected and T cells are engineered to treat a variety of B-cell malignancies. Although this technology is effective for blood cancers, its limited effectiveness against solid tumors due to poor infiltration against the complex tumor microenvironment has prompted researchers to look for a delivery system that will allow CAR-T cells easier access to cancerous cells [183]. Using injectable or implantable bio scaffolds for locoregional delivery has been successful, and codelivery of CAR-T cells with immunostimulatory molecules has improved long-term delivery into the tumor microenvironment. For instance, Grosskopf AK et al., in a mouse model, used a polymer-nanoparticle hydrogel (PNP) to deliver CAR-T cells with IL-15, both proximal and distal to tumors, potentially accessing solid tumors and curing them [184].

Combination therapy in one or more modalities can have a synergistic effect on the treatment of solid tumors. Hu et al. carried out one such study where they used CAR-T cells in combination with immune checkpoint inhibitors in a melanoma mouse model. Biodegradable hydrogel encapsulates CAR-T cells, targeting human chondroitin sulfate proteoglycan 4 (CSPG4.CAR) with nanoparticle-coated IL-15 and anti-PDL1 conjugated with human platelets in combination, allowing IL-15 to activate and proliferate CAR-T cells while blocking the PD1/PDL1 pathway to eradicate tumor cells [185]. Various delivery modalities have been used to effectively deliver and improve the access of CAR-T cells to solid tumors. Biomedically designed polymeric devices can provide effective access to incompletely resected or inoperable tumors, and the conjunction of soluble biomolecules and T-cell activation antibody ligands can achieve the multi-faceted promotion of antitumor activity against cancerous cells. 3D bio scaffolds such as polymerized alginate-collagen mimetic peptide matrices aided T cell migration to the tumor site, as did combining porous silica microparticles into matrices capable of encapsulating and releasing biomolecules for extended periods of time [186].

With new delivery technologies emerging for cancer immunotherapy, one has to carefully choose the appropriate method by which drug efficiency can be improved. Some of the delivery technologies are listed below with their advantages and disadvantages for cancer immunotherapy (Table 4).

## 5. Clinical Trails and Patents

Clinical trials are conducted using new delivery systems for cancers. To analyze, clinically translate and market personalized medication, rigorous research is required. Some of the clinical trials that have been studied to determine their effectiveness and safety are shown in below in Table 5.

Cancer drugs require novel delivery systems to make them effective and safe therapies. Recent anti-tumor therapies are designed in such a way they are efficacious in dealing complex tumor environment. Listed below in Table 6 are some of the innovative discoveries to combat cancer using various delivery techniques.

## 6. Challenges and Future Progress

New developments in understanding the cancer prognosis and novel therapeutic approaches have called for innovative delivery methods in administering anticancer drugs. Immunotherapy-based drugs are currently studied in various types of cancers; their effect on solid tumors is meager because the low infiltration of immune cells makes lower tumor immunogenicity, leading to an immunosuppressive tumor environment. Developing unique and novel drug delivery systems in combination with multiple cancer therapies would allow the treatment of solid tumors. The key issues such as the controlled release of drugs at the specific site, techniques to assess these delivery mechanisms and their effect on the cellular or molecular level are some of the constraints in developing a robust delivery system. In the past thirty years, cancer nanomedicines-based approaches have achieved progress in tackling the tumor microenvironment by understanding the enhanced permeability and retention, but still certain hurdles in clinically driven transition in developing and approving are needed to be addressed [197].

Many cancer nano formulations have certain drawbacks like off-target accumulation, stability, in vitro to in vivo correlation and fulfilling regulatory norms in bringing clinical translation is of some major issues [198]. Major challenges in developing drug delivery systems using nanoparticle include physiochemical characterization, large-scale production, developing low-toxicity nanoparticles and fulfilling the regulations in their successful release into the market [199]. CAR-T cell therapy has gained interest after breakthrough approvals recently, but still various clinical applications need to be resolved, with better cell engineering and genome-editing technologies to improve the efficacy and safety against various types of cancers. Despite promising results with delivery methods using extracellular vesicles, nanoparticles, scaffolds and cellular-based vehicles to deliver drugs against cancers, more insights into the mechanism of TME to effectively infiltrate and the evade immune system are needed for these treatments to reach their full potential in countering cancers.

A multicentric approach in developing oncological therapeutic research using novel drugs and delivery systems has gained popularity with the advancement of 3D-printing and personalized delivery digital devices. In the future, there will be significant progress in the development of nanorobots or implantable microchips that can deliver drugs and control tumor progress. The future onco-medicine developments require intelligent and robust multi-disciplinary approaches, where computer-based artificial intelligence and biotechnology should go hand in hand in developing intelligent nanorobotic-based drug carriers for delivering nanomedicines [200]. One has to carefully iterate their potential to impact on animals and environment, which needs to be considered before their approval for treatment.

## 7. Conclusions

Immunotherapy was developed in response to the ever-increasing research on cancer and understanding and the use of technologies to find an effective treatment for cancer. Because cancer is a complex disease, smart and intelligent delivery technologies must be developed to overcome the challenges of controlling its growth and elimination. To achieve successful cancer remission, novel strategies and therapy regimens will be tested in preclinical and clinical research. In this review, we discuss the various cancer treatment approaches that use drugs and biomaterials to exploit the immunological cascades against the tumor microenvironment. Despite limitations and challenges in developing technologies to increase drug delivery or efficacious results, combining one or more therapies with improved delivery technologies can result in an effective clinical translation. Precision targeting approaches with immunologically effective, low-toxicity technologies in cancer immunotherapy and delivery should translate to clinical implications and eventually benefit patients.

## Figures and Tables

**Table 1:** Cancer Immunotherapy Products on the Market Approved by the US FDA [17].

Product Name	Therapy	Type	Cancers Approved	Approved Year
Roferon-A	Recombinant IFNα2a	Cytokine	Hairy cell leukemia, follicular lymphoma, melanoma, Kaposi sarcoma	1986
Intron-A	Recombinant IFNα2b	Cytokine	Hairy cell leukemia, follicular lymphoma, melanoma, Kaposi sarcoma	1986
Aldesleukin	Recombinant IL-2	Cytokine	Melanoma and kidney cancer	1992
Sipuleucel-T	Autologous PBMCs activated with Recombinant human PAP–GM-CSF	Cell-Based Cancer Vaccine	Prostate cancer	2010
Ipilimumab	CTL A4 mAb	ICI	Melanoma	2011
Nivolumab	Anti PD-L1 (PD-L1 mAb)	ICI	Melanoma, lung cancer, malignant pleural mesothelioma, renal cell carcinoma, Hodgkin lymphoma, head and neck cancer, urothelial carcinoma, colon cancer, esophageal squamous cell carcinoma, liver cancer, gastric cancer, and esophageal or gastroesophageal junction cancer	2014
Pembrolizumab	Anti PD-L1 (PD-L1 mAb)	ICI	Melanoma, lung cancer, head and neck cancer, Hodgkin lymphoma, stomach cancer, cervical cancer, and certain types of breast cancer.	2014
T-Vec (Talimogene laherparepvec)	GE Oncolytic HSV1 with GM-CSF	Oncolytic Virus	Melanoma	2015
Atezolizumab	Anti PD-L1 (PD-L1 mAb)	ICI	Urothelial carcinoma, non-small cell lung cancer (NSCLC), triple-negative breast cancer, small cell lung cancer, hepatocellular carcinoma, and alveolar soft part sarcoma.	2016
Tisagenlecleucel	CD19-specific CAR-T cells	Adoptive cell therapy	B cell acute lymphocytic leukemia and non- Hodgkin lymphoma	2017
Axicabtagene ciloleucel	CD19-specific CAR-T cells	Adoptive cell therapy	Large B cell lymphoma	2017
Brexucabtagene autoleucel	CD19-specific CAR-T cells	Adoptive cell therapy	Mantle cell lymphoma (MCL) and acute lymphoblastic leukemia (ALL)	2020
Lisocabtagene maraleucel	CD19-specific CAR-T cells	Adoptive cell therapy	B cell non-Hodgkin lymphoma	2021
Idecabtagene vicleucel	B cell Maturation antigen (BCMA)	Adoptive cell therapy	Multiple myeloma	2021
Ciltacabtagene autoleucel	BCMA	Adoptive cell therapy	Multiple myeloma	2022
Opdualag	PD1 blocking and Anti-LAG-3	ICI	Melanoma	2022

**Table 2:** Route of administration for cancer immunotherapy.

Route of Administration	Advantages	References
Oral Administration	Simple and non-invasiveness Innovative strategies such as nanoionization, lipid-based formulations, permeation enhancers and gastroretentive dosage forms can be made available for effective delivery Applicable for multi cancer or metastatic treatments	[78,79,80]
Intravenous Administration	High bioavailability Low inter/intra patient variability Ease of delivery with nanomedicine and biomolecule-based formulations	[81,82,83]
Subcutaneous Administration	Controlled release of drugs Ease of injectability Future implantation like microchips and controlled release bioconjugates technologies are extensively researched	[84,85,86]

**Table 3:** Delivery techniques for cancer immunotherapy.

Delivery Technology	Types/Source	Cargo	Cancer Type	Reference
Nanoparticles	Liposomes	ErbB2/HER2 peptide	Renal carcinoma	[87]
		OVA	Thymoma	[88]
		ACT-cell-specific antibodies and Interleukin-2 (IL-2)	Melanoma	[89]
		Plasmid encoding telomerase-specific oncolytic adenovirus	Colorectal cancer	[90]
	Polymer	OVA and Hydroxychloroquine	Thymoma	[91]
		PLK1 inhibitor and PD-L1 antibody,	NSCLC	[92]
		IR780 and PD-L1 antagonist	Colorectal cancer	[93]
	Dendrimer	PD-L1 siRNA and IL-2 encoding plasmid DNA	HCC	[94]
	Inorganic nanocarriers	Vesicular stomatitis virus,	Colorectal cancer	[95]
		Adenovirus	Pancreatic cancer, Colorectal cancer	[96]
		mRNA-encoding OVA and R848	Melanoma	[97]
	RNA/DNA Technology	Anti-PD-1 antibody and CpG oligodeoxynucleotides,	Melanoma	[98]
		OVA	Melanoma	[99]
	Exosomes	Let-7a miRNA	Breast cancer	[100]
		EGFR nanobodies	Epidermal	[101]
		Cisplatin	Ovarian cancer and Hepatocarcinoma	[102]
	Nanovaccine	Peptide neoantigen (Adpgk) and R848 and CpG	Colorectal cancer	[103]
		cyclic dimeric guanosine monophosphate (CDG)	melanoma	[104]
Extracellular Vesicles	Dendritic cells	VEGF siRNA	Breast cancer	[105]
	Bone Marrow-Derived MSC	TRAIL	lung Cancer	[106]
	A549 Lung Carcinoma ells (Human)	Doxorubicin	Lung carcinoma	[107]
	B16-F10 melanoma cells (Mouse)	CpG DNA	Melanoma	[108]
	H22 Hepatocarcinoma cells (Mouse)	Doxorubicin, 5-FU	Hepatocarcinoma	[109]
Implantable Scaffolds	Collagen and HA cross-linking scaffold	GEM, poly(I:C)	Breast cancer	[110]
	PLG scaffold	GM-CSF, CpG-ODNs	Melanoma	[111]
	Hyaluronic acid scaffold	CAR-NK cells	Breast cancer	[112]
Injectable Scaffolds	Alginate Hydrogel	Celecoxib, PD-1 antibody	Melanoma, Breast cancer	[113]
	PEGylated poly(L-valine) hydrogel	TCL, poly(I:C)	Melanoma	[114]
	ROS-degradable hydrogel	GEM, PD-L1 antibody	Melanoma, Breast cancer	[115]
Cell-Based Delivery	Erythrocyte	Curcumin	Liver cancer	[116]
		Glucose oxidase, Tirapazamine	Colon cancer	[117]
		DOX	Lymphoma	[118]
	Cytotoxic T cells	Taxol	Gastric cancer	[119]
	NK cell	TCPP	Breast cancer	[120]
	Car-T Cells		Glioblastoma, hepatic colorectal metastases, peritoneal carcinomatosis, pleural mesothelioma, mesothelioma	[121]

**Table 4:** Advantages and disadvantages of delivery techniques in cancer immunotherapy.

Delivery Modalities	Immunotherapy Classes	Advantages	Disadvantages
Nanoparticles	Immune Checkpoint Inhibitors Cytokines Agonistic antibodies Engineered T cells	Surface functionalization with targeting agents Delivery to specific localities Cargo protection	Stability Off-target Drug Release Nanoparticle Toxicity
Extracellular Vesicles	Vaccines Cytokines Engineered T cells	Low Immunogenicity High Biodistribution rate Versatile drug carrier	Limited knowledge in mechanism of action Difficult to mass produce
Implantable Scaffolds	Vaccines Cytokines Engineered T Cells	Delivery of dendritic cells attractants and activation Controlled release agents Cargo protection Structural cues for cell	Requires surgery Probable rejection of loaded adjuvant
Injectable Scaffolds	Immune Checkpoint Inhibitors Cytokines Neoantigens	Minimal invasiveness Controlled Release Direct delivery to the tumor Controlled release of agents	Still under Research and Development Need more characterization Use of higher gauge needles
Cell-Based Delivery	Engineered T cells Adoptive T cell Therapy	High Affinity Binding High cell numbers Repetitive Killing possible	Individual manipulation of T cells Loss of reactivity during expansion Cross reactivity & “off-target” activation

**Table 5:** Current clinical trials for cancer immunotherapy by various delivery technologies.

Clinical Trial Identifier	Phase	Treatment	Therapy	Delivery Modalities	References
NCT00466960	II	Sargramostim and Paclitaxel Albumin-Stabilized Nanoparticle Formulation in Treating Patients With Advanced Ovarian Cancer, Fallopian Tube Cancer, or Primary Peritoneal Cancer That Did Not Respond to Previous Chemotherapy	Combined Therapy (Chemotherapy and Cytokine)	Nanoparticle	[187]
NCT02410733	I	Evaluation of the Safety and Tolerability of i.v. Administration of a Cancer Vaccine in Patients with Advanced Melanoma (Lipo-MERIT)	Vaccine	Liposome	[188]
NCT01753089	I	Dendritic Cell Activating Scaffold in Melanoma	Cell Therapy	Scaffold	[189]
NCT00103506	III	Study of DOXIL/CAELYX (Pegylated Liposomal Doxorubicin) and VELCADE (Bortezomib) or VELCADE Monotherapy for the Treatment of Relapsed Multiple Myeloma	Chemotherapy	Liposome	[190]
NCT02379845	II/III	NBTXR3 Crystalline Nanoparticles and Radiation Therapy in Treating Randomized Patients in Two Arms with Soft Tissue Sarcoma of the Extremity and Trunk Wall	Radiotherapy	Nanoparticle	[191]
NCT01052142	I	Safety Study of a Liposomal Vaccine to Treat Malignant Melanoma	Vaccine	Liposome	[192]
NCT00157209	IIb	Phase 2b Randomized Controlled Study of Tecemotide (L-BLP25) for Immunotherapy of NSCLC (Non-Small Cell Lung Cancer)	Vaccine	Liposome	[193]
NCT00924326	I/II	CAR T Cell Receptor Immunotherapy for Patients With B-cell Lymphoma	CAR-T		[194]
NCT01454596	I/II	CAR T Cell Receptor Immunotherapy Targeting EGFRvIII for Patients with Malignant Gliomas Expressing EGFRvIII	CAR-T		[195]
NCT01865617	I/II	Laboratory Treated T Cells in Treating Patients with Relapsed or Refractory Chronic Lymphocytic Leukemia, Non-Hodgkin Lymphoma, or Acute Lymphoblastic Leukemia	CAR-T		[196]

**Table 6:** Novel patents for cancer immunotherapy by various delivery technologies.

Patent Number	Inventors	Title
US20090010948A1	Fang Ping Huang, Yu Xiao Chen, Kwan Man	Anti-tumor vaccines delivered by dendritic cells devoid of interleukin-10
US20040156846A1	Wolfgang Daum, Gerald DeNardo, Diane Ellis-Busby, Alan Foreman, Douglas Gwost, Erik Handy, Robert Ivkov	Therapy via targeted delivery of nanoscale particles using L6 antibodies
WO2017151727A1	Zhen GU, Chao Wang, Yanqi YE	Enhanced cancer immunotherapy by microneedle patch-assisted delivery
US20160361268A1	Chih-Peng Liu, Ya-Chin Lo, Ming-Cheng Wei, Maggie LU, Shuen-Hsiang CHOU, Shih-Ta Chen, Hsiang-Wen TSENG	Intralymphatic delivery of hyaluronan nanoparticle for cancer metastasis
WO2011097384A2	Dapeng Zhou, Li Chun, Patrick Hwu	Tumor targeted delivery of immunomodulators by nanoplymers
US8785371B2	Rameshwar Patil, Eggehard Holler, Keith L. Black, Julia Y. Ljubimova	Drug delivery of temozolomide for systemic based treatment of cancer
US20160346204A1	Wenbin Lin, Chunbai He, Demin Liu	Nanoscale carriers for the delivery or co-delivery of chemotherapeutics, nucleic acids and photosensitizers
US9610250B2	Tarek M. Fahmy, Eric STERN, Richard A. Flavell, Jason Park, Alyssa Siefert, Stephen H. Wrzesinski	Nanolipogel vehicles for controlled delivery of different pharmaceutical agents
US20080044484A1	Boris Minev	Use of polymeric nanoparticles for vaccine delivery
US20040038406A1	Gretchen Unger, Beverly Lundell	Nanoparticle delivery systems and methods of use thereof

## Data Availability

All data needed to support the conclusions are present in the paper. Additional data related to this paper may be requested from the authors.
